# Is there a relationship between tonsil volume and the success of pharyngeal surgery among adult patients with obstructive sleep apnea?

**DOI:** 10.1016/j.bjorl.2021.12.002

**Published:** 2022-01-04

**Authors:** Silvia Matarredona-Quiles, Marina Carrasco-Llatas, Paula Martínez-Ruíz de Apodaca, Noelia Ortega-Beltrá, José Dalmau-Galofre

**Affiliations:** Hospital Universitario Doctor Peset, Department of Otolaryngology, Head and Neck Surgery, Valencia, Spain

**Keywords:** Obstructive Sleep Apnea, Palatine tonsil, Sleep-disordered breathing

## Abstract

**Objectives:**

Hypertrophic palatine tonsils play a role in the blockage of the upper airway, one of the known causes of Obstructive Sleep Apnea (OSA). Therefore, it is possible that there is an association between tonsil size and the success of pharyngeal surgery during OSA treatment. The main objective of this study was to evaluate the relationship between tonsil grade and volume, as well as to establish whether a relationship exists between tonsil size and the success rate of pharyngeal surgery (tonsillectomy and pharyngoplasty with barbed sutures).

**Methods:**

This retrospective study includes forty-four adult patients who underwent tonsillectomy and pharyngeal surgery with barbed sutures for the treatment of simple snoring and OSA between January 2016 and September 2019. Patients who had been previously tonsillectomized or those for whom tonsil volume measurement was lacking were excluded. All patients underwent a pre-operative physical exploration at the clinic exam room and a sleep study. Prior to surgery a Drug Induced Sleep Endoscopy (DISE) was performed. Tonsil volume was measured intraoperatively using the water displacement method. The same sleep study was repeated six months following surgery.

**Results:**

A significant correlation was found between tonsil grade and volume and between such measurements and the blockage observed at the level of the oropharynx during the DISE. Moreover, an association was observed between tonsil volume, but not tonsil grade, and the success of tonsillectomy and pharyngoplasty with barbed sutures. A tonsil volume greater than 6.5 cm^3^ was linked to success during pharyngeal surgery.

**Conclusion:**

A correlation exists between tonsil grade and tonsil volume. A bigger tonsil volume is associated with a greater success rate of oropharyngeal surgery during treatment of OSA.

**Level of evidence:**

Level 3, non-randomized cohort study.

## Highlights

• A positive correlation exists between tonsil grade and volume.
• Tonsil volume and surgical success were correlated.
• The volume of the tonsils of those patients whose surgery was deemed a success was greater (9.2 cm^3^ vs. 5.5 cm^3^).
• A tonsil volume greater than 6.5 cm^3^ was linked to success during pharyngeal surgery.

## Introduction

Obstructive Sleep Apnea (OSA) may have multiple causes that ultimately lead to a blockage of the Upper Airway (UA). This blockage is primarily located in the pharynx, palatine tonsils being structures that can affect such obstruction. Palatine tonsils size is usually assessed in a subjective manner through an oropharyngeal examination, or in an objective fashion via measurement of tonsil volume following extraction. Tonsil subjective size can be evaluated with Tonsil grading system from Friedman^1^ or Brodsky classification,^2^ which are comparable (Table 1).

**Table 1:** Comparison of tonsil grade definitions according to Brodsky and Friedman’s classifications.

Tonsil grade	From Friedman^1^	From Brodsky^2^
0	Absence of tonsillar tissue	Abscence of tonsilar tissue
1	Tonsils within the pillars	Tonsils were hidden in the pillars
2	Tonsils extended to the pillars	Tonsils were beyond the anterior pillar and between 25% and 50% of the pharyngeal space
3	Tonsils extended past the pillars	Tonsils were beyond the pillars but not to the middle and occupied >50% and up to 75% of the pharyngeal space
4	Tonsils extended to the midline	Tonsils occupied >75% of the pharyngeal space

The subjective evaluation of tonsil size is based on the airway space that remains free, rather than on the tonsil volume *per se*.^1^ Therefore, it is possible that structural variations in elements of the lateral pharyngeal wall may induce the under- or overestimation of the true size of the tonsils. Nevertheless, a relationship between tonsil grade and objective tonsil volume has been observed in several studies both in children^3^, ^4^, ^5^ and adults.^2^, ^3^, ^4^, ^6^, ^7^, ^8^ A link has also been established between tonsil size and the severity of OSA.^3^, ^7^, ^8^, ^9^, ^10^ Hence, it is entirely plausible that if tonsil size is one of the components contributing to the blockage of the airway, this parameter could correspond with the success of pharyngeal surgery. Indeed, multiple works have corroborated that subjective tonsillar hypertrophy relates to surgical success (tonsillectomy with or without pharyngoplasty).^11^, ^12^, ^13^, ^14^, ^15^

In theory, palatine tonsil size measured with subjective scales or objectively should be related to pharyngeal surgery success because tonsils are part of the pharyngeal structures that cause airway obstruction. Nevertheless, there might be no correlation between subjective and objective tonsillar size given that subjective tonsillar size is influenced by different anatomical variations.

Regarding the evaluation of the volume of the tonsils removed, there are different methods such as Archimedes, Cavalieri and Ellipsoid methods.^4^ Tonsillar volume can be calculated according to Archimedes’ principle by placing the removed tonsil into a syringe containing water.^4^ Also, it can be calculated using Ellipsoid formula by measuring the longitudinal, transverse and anteroposterior diameter.^4^ Cavalieri method allows estimating tonsillar volume by physical sectioning.^4^ There are other methods to estimate tonsil volume using Computed Tomography (CT), Magnetic Resonance (MRI) or hand held portable oral camera with 3D imaging.^16^ But this type of technology is not readily available in a clinic setting.

The present study aimed to evaluate the relationship between tonsil size observed during oropharyngeal examination (tonsil grade) and objective tonsil volume (tonsil volume) in adult patients with sleep-related breathing disorders. Additionally, this work intended to elucidate whether a link exists between the aforementioned parameters and oropharyngeal surgical success. Furthermore, we aimed to calculate the tonsillar volume necessary to predict a successful oropharyngeal surgery during OSA treatment.

## Methods

Retrospective study involving adult patients (over 18-years of age) who underwent pharyngeal surgery for the treatment of simple snoring and OSA between January 2016 and September 2019. Simple snoring patients were included in the study with the aim of relying on a greater rating of the Apnea-Hypopnea Index (AHI) whenever correlating AHI with tonsil size. Thus, we avoided exclusively including patients with a pathological AHI, which would have been less representative of the general population.

Those patients who had previously undergone tonsillectomy or for whom the measure of their tonsils volume was unknown were excluded. Patients with a body mass index (BMI) higher than 36 kg/m^2^ are not considered candidates for pharyngeal surgery in OSA, likewise obese patients were not included. Forty-four patients constituted the study population. All patients were evaluated and treated by the same sleep surgeon.

During the pre-surgical physical exploration, the subjective tonsil grade was assessed using Tonsil grading system from Friedman et al.^1^: Size 0, absence of tonsillar tissue; Size 1, within the pillars; Size 2, extended to the pillars; Size 3, extended past the pillars; Size 4, extended to the midline. In cases of subjective tonsil asymmetry, the highest tonsil grade among both tonsils was assigned.

Prior to surgery, the cervical circumference was also measured to estimate the potential effect of gathering fat at the cervical level along with narrowing at the level of the lateral pharyngeal walls, which could otherwise lead to an overestimate of the subjective tonsil size. A fiber endoscopic exploration was performed to all patients at the clinic exam room in order to assess airway obstruction during wakefulness at the level of the tongue base using Woodson’s classification.^17^ Before surgery, Drug Induced Sleep Endoscopy (DISE) was carried out; and the type and degree of blockage was classified according to the modified VOTE (velum, oropharynx, tongue base and epiglottis) classification.^18^

The pharyngeal surgery performed consisted of tonsillectomy and pharyngoplasty with barbed sutures. However, variations in the technique took place depending on each patient’s anatomical features. Hence, a partial uvulectomy was performed in one case and removal of supratonsillar fat was performed in 32 patients (72,7%). Supratonsillar fat removal was performed in subjects suffering from overweight or grade I obesity.

The objective tonsil volume was calculated during surgical intervention following tonsils excision by the water displacement method (Archimedes’ principle). Accordingly, tonsils were placed in a 20 cm^3^-syringe filled with 10 cm^3^ of serum. The final volume obtained minus the initial 10 cm^3^ corresponded to the volume of the removed tonsils.

All patients underwent a pre-surgical sleep study (polysomnography or cardiorespiratory polygraphy). The same test was repeated six months after the surgical intervention.

Pre- and post-surgical additional clinical data were included, such as the weight and the Body Mass Index (BMI). Surgery was deemed a success whenever the AHI was reduced >0% and the post-surgical AHI was lower than 20 events/h (Sher criteria).^19^

Using GRANMO© freeware, a minimum sample size of 20 patients was calculated for significance purposes. An alpha risk of 0.5 and a beta risk of 0.2 in a bilateral contrast were accepted, considering a correlation coefficient of 0.6 and a lost to follow-up rate of 0%, since patients with missing data were not included.

Statistical analyses were performed in IBM© SPSS© (Statistical Package for the Social Sciences), version 24 for OS X©. Basal characteristics of patients were evaluated using a descriptive study, with the mean and standard deviation for continuous variables and frequencies for qualitative variables.

Kruskal–Wallis test was used to assess the relationship between tonsil grade and the continuous variables (age, BMI, AHI, cervical circumference, tonsil volume). This same method was also employed to evaluate the association between tonsil volume and the qualitative variables (Woodson’s classification, degree of obstruction at the veil of the palate during DISE, degree of obstruction at the oropharynx during DISE, degree of obstruction at the base of the tongue during DISE). Spearman’s rho test was used to study the correlation between tonsil volume and the continuous variables (age, BMI, AHI, cervical circumference). The relationship between tonsil grade and the qualitative variables (Woodson’s classification, degree of obstruction at the veil of the palate during the DISE, degree of obstruction at the oropharynx during DISE, degree of obstruction at the base of the tongue during DISE, morphology of collapse) was determined with Pearson’s Chi-Squared test.

After testing the normality, tonsil volume was compared between cases of surgical success and failure using the Mann–Whitney *U* test, and tonsil grade was compared between cases of surgical success and failure using the Chi-Squared test. A ROC (receiver operating characteristic) curve was used to predict which minimum tonsil volume be considered surgical success. For all tests, significance was set at a *p*-value of <0.05.

## Results

Forty-four patients suffering from simple snoring or OSA who underwent tonsillectomy and pharyngoplasty with barbed sutures constituted the study population. Among them, 90.9% were men with an average age of 37.9 ± 10.8 years, BMI of 28.1 ± 4.2 kg/m^2^, and AHI of 43 ± 29.5/h (4.4–126.1/h). Within the study population, 13.6% suffered from mild OSA, 20.5% from moderate OSA, 61.5% from severe OSA, and 4.5% from simple snoring. Data collected during the physical exploration during wakefulness and during the DISE are presented in Table 2, as well as the mean tonsil volume recorded following tonsillectomy.

**Table 2:** Data collected during the oropharyngeal exploration during wakefulness, during DISE, and the objective tonsil volume recorded in patients who underwent pharyngoplasty with barbed sutures (n = 44).

	Mean ± SD or %
**Tonsil grading system from Friedman (%)**	
0	0
1	18.2
2	20.5
3	59.1
4	2.3
**Woodson’s classification (%)**	
1+	15.9
2+	52.3
3+	27.3
4+	4.5
**Pre-surgical cervical circumference (cm)**	40.9 ± 3.1
**DISE: degree of velum obstruction (%)**	
0	8.3
1	2.8
2	88.9
**DISE: degree of oropharyngeal obstruction (%)**	
0	8.1
1	8.1
2	83.8
**DISE: degree of tongue base obstruction (%)**	
0	61.1
1	19.4
2	19.4
**Tonsil volume (cm^3^)**	8.2 ± 3.9

Table 2 shows the sample distribution according to the subjective tonsil grade evaluated with Friedman’s classification (0–4), the obstruction of the airway during wakefulness at the level of the tongue base using Woodson’s classification (1–4), the cervical circumference measured in cm, and the degree of obstruction of the airway during Drug Induced Sleep Endoscopy (DISE) according to a modified VOTE (velum, oropharynx, tongue base and epiglottis) classification: 0, no obstruction; 1, partial obstruction (vibration); 2, complete obstruction (collapse >75%).

A positive correlation was found between tonsil grade and tonsil volume (*p* = 0.000) (Table 3). Thus, the greater the tonsil grade, the bigger the tonsil volume measured after the tonsillectomy. We also observed a greater collapse at the level of the oropharynx during DISE as tonsil grade got higher (X^2^ = 0.024). Tonsil volume was linked to age (*p* = 0.003), to the degree of obstruction at the level of the oropharynx during DISE (*p* = 0.034) and to the cervical perimeter (*p* = 0.05). A significant association to any of the remaining analyzed factors was not observed (Appendix 1).

**Table 3:** Mean tonsil volume distribution according to tonsil grading system from Friedman, modified Mallampati index also known as Friedman tongue position and Woodson’s classification.

	Tonsil volume (Mean ± SD) (cm^3^)	*p*
**Tonsil grading system from Friedman**		0.000
1	3.86 ± 1.46	
2	6.5 ± 2.35	
3	9.8 ± 3.8	
4	10 ± 0	
**Modified Mallampati index**		0.001
1	–	
2	9.3 ± 4.2	
3	6.7 ± 2.5	
4	8.2 ± 2.6	
**Woodson’s classification**		0.447
1+	5.41 ± 2.04	
2+	7.59 ± 3.05	
3+	9.17 ± 4.75	
4+	5.5 ± 0.71	

After surgery, the mean AHI was significantly reduced to 14.2 ± 15.4/h (*p* = 0.000) while the (mean) BMI increased to 30 ± 5.6 kg/m^2^ (*p* = 0.000). 14% of patients were healed (AHI < 5/h), 25.6% were mild OSA, 27.9% moderate OSA, and 31.8% severe OSA. According to Sher criteria, surgical success was 65.6%. Tonsil volume was correlated with surgical success (*p* = 0.019), but this was not the case neither with the tonsil grade, nor with the degree of obstruction at the veil of the palate, oropharynx, and the tongue base (*p* = 0.783) observed during DISE (Table 4). We also did not find a relationship between surgical success and the morphology of collapse (antero-posterior, lateral or concentric) at the level of the palate (*p* = 0.093) or the tongue base (*p* = 0.502).

**Table 4:** Factors correlated with surgical success.

	*p*
Tonsil grading system from Friedman	0.392
Tonsil volume	0.019
DISE: degree of velum obstruction	0.195
DISE: degree of oropharynx obstruction	0.169
DISE: degree of tongue base obstruction	0.783

**Figure 1:**
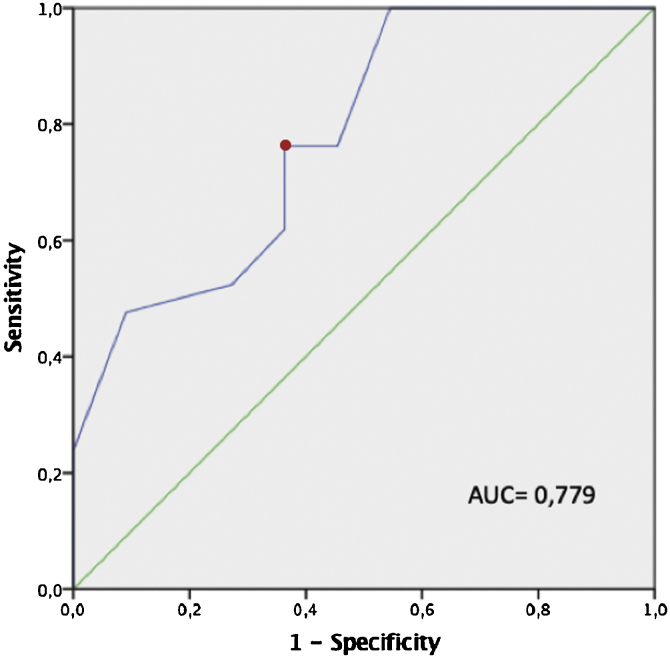
ROC curve of tonsil volume and surgical success. Fig. 1 shows a ROC (Receiver Operating Characteristic) curve (blue) in which we observe that a tonsil volume of 6.5 cm^3^ (red dot) is the tonsil volume with the highest sensitivity (76.2%) and specificity (63.3%). A volume of 6.5 cm^3^ is the cut-off value with the highest sensitivity and specificity to detect subjects for whom pharyngeal surgery during OSA treatment should be the most successful.

Among those patients whose pharyngeal surgery was considered successful, a significantly greater tonsil volume was observed (tonsil volume of 9.2 cm^3^ vs. 5.5 cm^3^; *p* = 0.009). The ROC curve estimated that a tonsil volume greater than 6.5 cm^3^ was linked to success during pharyngeal surgery (sensitivity, 76.2%; specificity, 63,3%) (Fig. 1).

## Discussion

The present study found a positive correlation between tonsil grade and tonsil volume in adult patients suffering from simple snoring and OSA. The degree of obstruction at the level of the oropharynx during DISE and tonsil grade and volume were also observed to be positively correlated. Tonsil grade was not influenced by the BMI or the cervical perimeter. Contrary to our expectations, we did not find any correlation between tonsil size, either objective or subjective, and the OSA severity. Nevertheless, based on our data, tonsil volume was deemed an adequate proxy for surgical success, since the greater the volume of the tonsils, the higher the percentage of patients who underwent a successful tonsillectomy and pharyngoplasty with barbed sutures. According to our results, a tonsil volume greater than 6.5 cm^3^ can anticipate surgical success. Moreover, we found a significant difference regarding the mean tonsil volume between patients whose surgery was considered successful and those whose surgery was regarded as failure.

In agreement with previous studies, a correlation between subjective tonsil grade and objective tonsil volume was observed.^2^, ^4^, ^5^, ^6^, ^7^, ^8^ However, opposite to previous works, we did not find a correlation between tonsil grade and BMI and cervical perimeter.^2^, ^7^ Such observation is relevant, as the assessment of the tonsil size in a subjective manner at the clinic exam room depends on the space occupied by the tonsils in the oropharynx. Such a space could be affected by the increase in soft tissues which could, in turn, medialize the tonsils giving the impression of a tonsil grade greater than in reality. The aforementioned rise in soft tissues at the level of the lateral pharyngeal walls may be a consequence of excess fat gathering at the said location, among others. Therefore, both BMI and cervical perimeter are factors that could become associated to an increase of soft tissues in the airway. The exclusion of morbid obese patients in our sample size could be an explanation for the absence of relation in our study.

In our study, we did not observe any association between tonsil size and AHI. As recorded in the systematic review by Nolan et al.^10^ regarding the relationship between tonsillar hypertrophy and the obstruction of the UA in children, eleven studies reported a significant association between the subjective tonsil size and the AHI. However, nine studies showing superior evidence did not describe such link.^10^ Among all of them, Howard et al. were the only ones who observed a positive correlation between the AHI and their measured objective tonsil volume, but they did not report any relationship between the subjective tonsil size and the AHI.^20^ The majority of studies which have analyzed such association in adult patients have confirmed a positive relationship between the subjective tonsil size and the severity of OSA (inferred by the AHI or by the oxygen desaturation index),^3^, ^7^, ^8^, ^9^ and between the objective tonsil volume and the severity of OSA.^3^, ^7^, ^8^ Still, the abovementioned association has not been demonstrated in more heterogenous groups of patients.^6^ This evidences the complexity and heterogeneity of the physiopathology of sleep apnea and the importance of considering all different phenotypes of this disorder, since AHI by itself is not enough to encompass the full spectrum of this syndrome.^21^

Regarding the potential influence of tonsil size on surgical success, previous studies have demonstrated the link between subjective tonsillar hypertrophy and the success of surgery (tonsillectomy with or without pharyngoplasty).^11^, ^12^, ^13^, ^14^, ^15^, ^22^ Yet, few have shown the relationship between tonsil volume and surgical success like we have in the present work. Tschopp et al. reported that higher tonsil volume and tonsil grade were related to a greater decrease of the AHI following surgery.^22^ Furthermore, we have estimated the minimum tonsil volume (i.e., 6.5 cm^3^) linked to surgical success, previously unreported in the literature. Besides, given that the tonsil volume of patients for whom surgery was considered successful averaged 9.2 cm^3^ compared to the mean 5.5 cm^3^ of those patients whose surgery failed, we cannot help but wonder whether pharyngoplasty adds extra benefits on top of tonsillectomy alone.

The fact that in patients with a tonsil volume smaller than 5.5 cm^3^ surgical success rate was reduced does not mean that oropharyngeal surgery cannot be successful for such patients or for those with small tonsils. Other prospective studies including patients who had been tonsillectomized in advance and selected using DISE showed a success rate for pharyngoplasty of approximately 84%, performing pharyngoplasty techniques with barbed sutures.^23^ Moreover, Chiu et al. observed a significant improvement of the AHI with a modified uvulopalatopharyngoplasty without tonsillectomy in patients with small tonsils (grade 0–2).^24^

The present study has some limitations. Despite data being collected in a prospective fashion, they were analyzed retrospectively. All patients were evaluated and treated by the single and not blinded sleep surgeon. Although the sample size was small, we included more than twice as many participants as needed in terms of statistical power to detect significant differences. Most patients were men, which limits the generalization of the results. Patients with a BMI > 36 kg/m^2^ were not considered candidates for surgery and, therefore, were not included in this study, our results not being applicable to this group of patients. Even if a pharyngoplasty with barbed sutures was performed to all patients, the technique employed was not exactly the same in all instances, since minor variations took place depending on each patient’s anatomy, which may have potentially affected the obtained results too, nevertheless we believe that this possibility is low because a partial uvulectomy was performed in a single patient and supratonsilar fat excision in overweight or obese patients does not seem to affect the results in our hands.^25^ Measurement of tonsil volume using the water displacement method may not reflect their true volume in cases in which the tonsils have absorbed liquid, but a more reliable method remains unknown to date. Notwithstanding, such eventuality is highly unlikely as the measurement of the tonsils takes just a few seconds and these are not submerged in any liquid prior to measurement.

## Conclusion

Tonsil grade and tonsil volume are correlated, and, in addition, they are both associated with the degree of obstruction observed at the level of the oropharynx during DISE. Tonsil volume was found to be linked to the success rate of tonsillectomy and pharyngoplasty with barbed sutures. Furthermore, a tonsil volume greater than 6.5 cm^3^ was linked to success of oropharyngeal surgery during OSA treatment. Further research is needed to determine if pharyngoplasty would be beneficial for patients with a tonsil volume higher than 9.2 cm^3^; as well as future studies comparing the differences in success for pharyngoplasty between patients who have been tonsillectomized in advance and those who have not undergone this procedure.

## Authors’ contributions

Conceptualization, Marina Carrasco-Llatas; methodology, Silvia Matarredona-Quiles; data collection, Paula Martínez-Ruiz de Apodaca, Noelia Ortega-Beltrá, Silvia Matarredona-Quiles and Marina Carrasco-Llatas; formal analysis, Silvia Matarredona-Quiles and Paula Martínez-Ruiz de Apodaca; resources, José Dalmau Galofre; writing-original draft preparation, Silvia Matarredona Quiles; writing-review and editing, Marina Carrasco Llatas and Paula-Martínez Ruiz de Apodaca. All authors have read and agreed to the published version of the manuscript.

## Funding

This research did not receive any specific grant from any funding agency in the public, commercial or not-for-profit sector.

## Conflicts of interest

The authors declare no conflicts of interest.
