# Supplementary material for: Is there a relationship between tonsil volume and the success of pharyngeal surgery among adult patients with obstructive sleep apnea?
Source: Braz J Otorhinolaryngol. 2022 Jan 4;88(Suppl 5):S156–61. doi: 10.1016/j.bjorl.2021.12.002 (PMC9801021; doi:10.1016/j.bjorl.2021.12.002)
Supplement: Supplementary file 1 [file mmc1.docx]

BJORL-D-21-00592 – Supplementary Material

**Appendix** **1** Variables without correlation with tonsil grade and volume.

| **Variables** | ***p*** |
| --- | --- |
| Tonsil grade |  |
| Age | 0.205 |
| BMI | 0.305 |
| AHI | 0.257 |
| Cervical circumference | 0.441 |
| Woodson’s classification | 0.672 |
| DISE: Degree of Velum obstruction | 0.463 |
| DISE: Degree of Tongue base obstruction | 0.371 |
| Tonsil Volume |  |
| BMI | 0.374 |
| AHI | 0.994 |
| Woodson’s classification | 0.447 |
| DISE: Degree of Velum obstruction | 0.148 |
| DISE: Degree of Tongue base obstruction | 0.589 |
